# Value-based medicine in oncology: the importance of perspective in the emerging value frameworks

**DOI:** 10.6061/clinics/2018/e470s

**Published:** 2018-11-27

**Authors:** Alessandro Gonçalves Campolina

**Affiliations:** Centro de Investigacao Translacional em Oncologia, Instituto do Cancer do Estado de Sao Paulo (ICESP), Hospital das Clinicas HCFMUSP, Faculdade de Medicina, Universidade de Sao Paulo, Sao Paulo, SP, BR

**Keywords:** Neoplasms, Drug Costs, Decision Support Techniques, Clinical Decision-Making, Medical Oncology, Resource Allocation

## Abstract

Recently, professional and healthcare-related entities have launched frameworks designed to assess the value of cancer innovations in multistakeholder decision processes. Among the most visible entities that propose and implement value frameworks in oncology are the European Society of Medical Oncology (ESMO), the American Society of Clinical Oncology (ASCO), the Memorial Sloan Kettering Cancer Center (MSKCC) and the National Comprehensive Cancer Network (NCCN). However, these value frameworks have been criticized for conceptual inconsistencies, inability to include a greater variety of value criteria, and inadequate explanation of the uncertainty approach used in the modeling process. On the other hand, Multi-Criteria Decision Analysis (MCDA) is a set of methods and processes that allow the multiple criteria involved in a decision to be explicitly addressed. This approach allows the identification of relevant decision criteria, gathering of evidence based on scientific literature, attribution of weights to the criteria and scores to the evidence raised, and aggregation of the weighted scores to constitute a global metric of value. The purpose of this article is to review the main features of these value frameworks in oncology and the importance of perspective for framework readiness to support healthcare decision-making based on MCDA methodology.

## INTRODUCTION

International estimates have shown that deaths from oncological disease ranked second among the different causes of death, totaling 8.2 million deaths in 2012. A significant increase in new cases, approximately 70%, is expected over the next two decades 1.

In Brazil, in the 2016-2017 biennium, the incidence of new cases of cancer was estimated at approximately 600,000, of which 420,000 were considered nonmelanoma skin cancer. The epidemiological profile observed resembles that of Latin America and the Caribbean, where prostate cancers (61 thousand) in men and breast (58 thousand) in women are the most frequent 2.

Because cancer is an emerging issue in industrialized and nonindustrialized countries, pharmaceutical companies are investing heavily in oncological research, and many new and effective treatments are being developed. Together with other economic and demographic trends, however, this emphasis causes anticancer drugs to place an increasing strain on global healthcare budgets 3.

In recent years, escalating drug prices have alarmed healthcare professionals and led to concerns about the government's role in regulating prices, and it has been suggested that, in some instances, drug launch prices may not be proportional to the health benefit they provide 3. Thus, many initiatives aimed at measuring and communicating the value of new technologies have been proposed as decision support alternatives in this context of increasing spending on healthcare technologies 4.

Some breakthroughs have been recognized. First, the development of frameworks for the evaluation of diagnostic and therapeutic technologies has been considered promising. Concerns about the progressive increase in drug costs are to be expected in a scenario of demographic and epidemiological transition, but justifications based exclusively on research, development and production costs by the pharmaceutical industry could be considered an inadequate strategy. On the other hand, a change in focus to prioritize what the general population considers therapeutic value, that is, what is truly desired from a healthcare intervention, could not only encourage the industry to produce products with higher added value but also stimulate innovation.

For the most part, value considerations could be made without the aid of formal decision support systems using intuitive approaches. However, when multiple factors may be implicated in healthcare decisions and outcomes – for example, in oncology, many choices are complex and involve several therapeutic options that imply varying degrees of preference for patients and healthcare providers – using such an approach can contribute to the decision-making process. Some of the key contributions of a decision support system are to enable a patient-centered approach, facilitate deliberative processes, assist in accessing relevant evidence and improve the effectiveness of decision-making.

The purpose of this article is to review the main characteristics of 4 of the most notable value frameworks in oncology and the importance of perspective for framework readiness to support health care decision-making among the intended stakeholders, using the Multi-Criteria Decision Analysis (MCDA) methodology as a reference.

### Value and perspective

Value is a multidimensional concept encompassing different aspects such as utility, social meaning, and emotional, spiritual and monetary significance. A wide range of disciplines have proposed varying sets of elements to consider what constitutes “value” 5.

Health economists define value based on what individuals would be willing to pay to access healthcare or other related services. Considering microeconomic principles, the idea of “willingness to pay” needs to consider the “opportunity cost”, that is, how much benefit people are willing to give up to obtain these extra health benefits 6,7. Despite the intrinsic relationship between value and efficiency, this relationship may not be clear with respect to healthcare interventions, especially for new technologies. For healthcare economic evaluation, the “net value” of a new intervention is an expression of the willingness to pay for health benefits in terms of the opportunity cost of the necessary resources to obtain the health benefit. In general terms, achieving “economic efficiency” is to maximize the value obtained with the invested resource in relation to other possible allocations of the same resource 8-10.

However, the perspectives of individuals and organizations involved in a decision-making process can impact certain value attributes in an evaluation process. The importance of perspective is increasingly being considered in the recently proposed value frameworks in oncology 11 (Table 1). What are the appropriate criteria or attributes of value? How could we obtain weights for the criteria considered by different stakeholders? How should these criteria be combined to obtain a general score? As these frameworks highlight, the particular “perspective” considered is a central question.

Notably, guidelines for cost-effectiveness analysis (CEA) emphasize the importance of clearly specifying from which perspective the analysis is being conducted 7,12,13. Different results can be obtained if this point of view is that of the health plan payer, the patient, the managers, the provider, the manufacturer of the technology, the specialties societies, or the general society. A sound healthcare economic evaluation could be conducted from any of these stakeholders' perspectives, depending on the scope and context of the analysis.

In CEA, it has been recommended that at least 2 perspectives should be considered, that of the health sector and that of society as a whole. However, any of the above perspectives could be considered individually 12,13.

**Figure 1:**
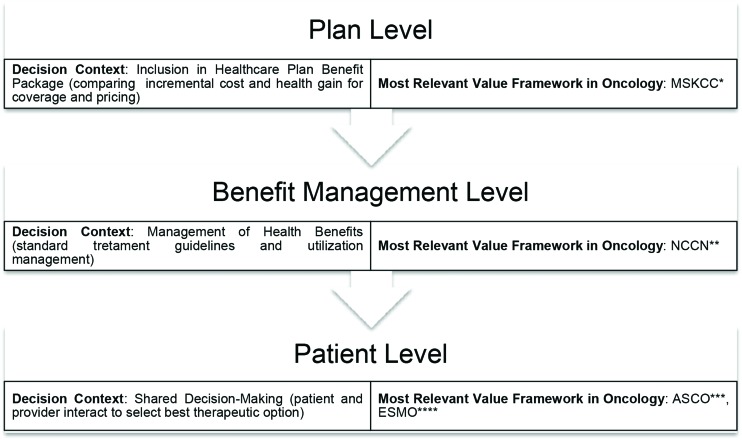
Decision Contexts and Value Frameworks in Oncology. Source: Schnipper, Davidson, Wollins, Blayney, Dicker, Ganz, et al. (2016), Cherny, Sullivan, Dafni, Kerst, Sobrero, Zielinski, et al. (2015), The Memorial Sloan Kettering Cancer Center (2016), National Comprehensive Cancer Network (2016). *Memorial Sloan Kettering Cancer Center; **National Comprehensive Cancer Network; ***American Society of Clinical Oncology; ****European Society of Medical Oncology.

Table 1 presents four recently developed value framework proposals. The difference in perspectives and contexts is striking among them, although they have been developed from the same normative view (Figure 1).

As summarized in Table 1, in general, different stakeholders may have more or less broad perspectives, depending on the context in which they are inserted. For example, a patient may care about health benefits only if he or she is in a context of “zero out of pocket” costs. A provider may be concerned only with the scarce medical resources required for the provision of a service, without worrying about the nonmedical costs borne by the patient. An employer may consider only costs related to absenteeism or presenteeism. An individual hesitating to acquire a new health insurance plan may worry about the costs and benefits of broader coverage. In general, all these perspectives are not broad enough to encompass all the aspects involved in the costs and effects of a healthcare technology. Despite the bias of each perspective, the important factor to the decision-maker is considering what might be lacking from their own perspective and how to make it as broad as possible if the goal is to maximize social well-being. In this sense, the great challenge would be how to face hard decisions considering multiple perspectives.

In a market society, using the two hats of the subscriber and the potential patient, the individual can make decisions based on the vision of these two different agents, who asymmetrically prioritize the costs and benefits of interventions, aided by the varying perspectives. Meanwhile, in universal healthcare systems, the individual can be seen as a collective or multi-stakeholder agent using two or even more hats. As a result, to assist such decisions, value frameworks should reasonably support stakeholders with divergent perspectives, considering that conflicts may exist and that trade-offs need to be made based on what each of the parties considers as an acceptable benefit.

### Value frameworks in oncology

Recently, to support multistakeholder decision-making, a number of healthcare-related and scientific societies have launched frameworks designed to assess the value of oncology therapies. These frameworks vary in terms of their concept of value, target audience, methodology, and stage of development 14-18.

Although healthcare organizations differ in their objectives, scope of activities and methodological approaches, the idea of implementing value assessment frameworks has been pursued by some of them. Among the most prominent entities, we can quote the European Society of Medical Oncology (ESMO), the American Society of Clinical Oncology (ASCO), the Institute of Clinical and Economic Research (ICER), Memorial Sloan Kettering Cancer Center (MSKCC) and the National Comprehensive Cancer Network (NCCN). Currently, these entities have proposed some of the most widespread value frameworks to date.

The four oncology value frameworks, reviewed in this study, present notable variations related to the type of decision being made. Decisions related to the coverage, access and price of new technologies are some of the most frequently considered. Some applications have also been concerned with supporting the construction of appropriate care lines and facilitating the shared decision-making process.

Table 1 presents a brief description of each of the value frameworks identified in oncology. Figure 1 presents a general characterization of these frameworks in terms of decision context and perspectives adopted. These two aspects can influence the definition of value adopted, insofar as they establish how the benefits and costs will be measured 4.

These new evaluation approaches that are not based on quality-adjusted life years (QALYs) have been criticized for having conceptual inconsistency by not including many of the relevant value criteria for healthcare decisions and for not adopting well-established procedures to address uncertainty in the valuation process. For example, some argue that since oncology-oriented frameworks are methods that have not been sufficiently tested, input and output choices are confusing, the approach is not patient-oriented, and the overall process is limited to considering more narrow aspects of the healthcare system, such as available pharmacological options. Specifically, in relation to the ASCO framework, several important components of value are omitted, arbitrary methods for weighting criteria and eliciting stakeholders' preferences are adopted, and costs of care are not considered. Furthermore, it has also been criticized for its lack of well-established measurement properties 19-22.

Like any healthcare measurement instrument, value assessment frameworks should demonstrate validity (measuring what it intends to measure) and reliability (consistency in different applications) 22,23. Different value frameworks showed convergent validity and good agreement in a comparison of applications made by different evaluators 23. However, Sorenson et al. observed important limitations in these frameworks. The ASCO framework did not involve patients in model design and value assessment, excluding the patient's voice from the decision process. The other frameworks offered only limited access to the economic model underlying the analyses 13.

Each of the frameworks has considerable limitations, which must be taken into account when interpreting its outputs. Whether the different approaches will converge in the future remains to be seen, but harmonization would help to limit confusion and aid stakeholders in making informed decisions in cancer patient care.

Notably, however, treatment “value” is an indefinable target, and there is no agreement regarding the domains that truly matter, how they should be incorporated into the tool (*e.g.,* additive *vs* multiplicative scoring), or how much weight should be given to each 4,24.

### Multicriteria decision analysis (MCDA)

Thus, some healthcare organizations have proposed guidelines that should guide the construction and application of value frameworks 25,26. These proposals focus on some principles that should guide future studies, including transparent processes for the development of frameworks, analytical models and reports; patient-centered care and focus on customizing the decision; access to high-quality scientific evidence; incorporation of a broad view of the effects; specification of indications of use and relevant applications; and inclusion of a wide range of healthcare interventions.

For population-level decisions, CEA and cost-per-QALY analysis have been recommended as a basis for structuring the underlying value constructs from the healthcare system perspective. Elements of value that are not typically captured in cost or QALY estimates may be considered in future decision analyses. In this sense, some of the most relevant elements are productivity, adherence, value of hope, equity concerns and adjustments for severity of disease to compensate for gains in utility.

**Figure 2:**
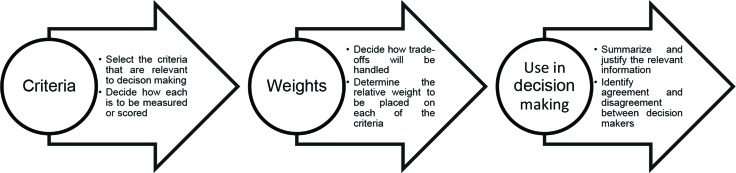
An Overview of the Multi-Criteria Decision Analysis (MCDA) Process. Source: Campolina (2017).

As the term implies, MCDA involves a set of methods and processes that allow the multiple criteria involved in a decision to be explicitly addressed. In this process, clarification of the criteria to be considered for the stakeholders involved and a report of the results of the decisions in a more transparent way are fundamental. Thus, in the MCDA methodology, criteria are identified, a score is attributed to each criterion to reflect its relative importance according to the different perspectives indicated in the process, evidence is systematically raised, scores are attributed to the performance of the evaluated technologies, and finally, the weighted scores are aggregated according to the preference of the participants to constitute a global metric of value 25 (Figure 2).

Thus, MCDA means considering a broad set of decision factors that go beyond the clinical benefits of a new healthcare technology to include other elements of value, such as burden of illness, equity, financial impact, ethical aspects, level of innovation, and environmental impact.

The first step in conducting MCDA is to identify factors that are relevant to the decision process. These factors can be identified from the scientific literature or through consultations with experts in the area of interest of the technologies evaluated. The second step involves weighing these factors based on what different stakeholders understand as relevant to the decision. Several methodologies may allow interviews with stakeholders to determine the relative importance of the criteria. Some of these methodologies are attractive for simplicity (such as point allocation exercises), and others are more sophisticated, involving a greater overload on the participating individuals (such as conjoint approaches). In the third step, the performance of each of the technologies can be compared based on the different factors, allowing the construction of a ranking system 26. Table 2 describes the eight steps in the implementation of MCDA, based on a value measurement approach, as recommended by the International Society For Pharmacoeconomics and Outcomes Research (ISPOR) Task Force for best practice in MCDA.

One factor that hinders the application of MCDA for Health Technology Assessment (HTA) methodologies is the great variability of existing approaches, which makes the existence of a standard approach difficult to establish. As a consequence, comparison of MCDA studies is extremely challenging. Another difficult aspect is the use of MCDA in a logic of budget constraint optimization, which would imply translating the value scores derived by MCDA into monetary values 27.

However, MCDA allows the use of approaches such as the Paraconsistent Decision Method (PDM), which is able to synthesize objective information (from previous studies) and subjective information (from the value judgments of experts in the area of knowledge), in addition to allowing the incorporation of conflicting, vague, and incomplete information that results from the limitations of the published studies or the variation in stakeholders perspectives 27.

Some practical aspects for the implementation of this type of modeling can be highlighted as follows: train all committee members in the use of MCDA and make facilitators available to assist in the use of the techniques in the decision process; select appropriate methods for data capture (questionnaires, printed or computerized forms, etc.) and aggregation (specific software); enable the exploration of the models to ensure the robustness of the criteria, which can be done in real time or between committee meetings; and finally, allow the outputs of the models to be visualized throughout the discussions and incorporated into the documentation of the report, together with the final recommendations.

### Conclusion

Finally, emerging challenges can be found in relation to the use of value criteria by oncology frameworks. Even though the emerging frameworks represent attempts to capture values that are important to many stakeholders, they are not always logical or consistent with the principles of decision theory. In part, these inconsistencies derive from problems of which perspective has been taken into account (patients, managers, healthcare professionals, etc.) and how these different and conflicting perspectives could be aggregated. In addition, given the lack of use of economic evaluation, value frameworks in oncology are not consistent with this basic recommendation for population-level decisions.

Healthcare decision-making that occurs in the absence of objective evaluation processes may result in discrepancies in how the importance of values and criteria should be considered, based on different perspectives. In this sense, the use of explicit approaches, such as MCDA, can facilitate the mediation of conflicts and optimize the participation of different stakeholders.

## Figures and Tables

**Table 1:** Value Frameworks in Oncology

	Year Established	Decision Context	Perspectives	Target Audience	Criteria and Attributes of Value	Conceptual Basis	Strengths
ASCO (14)	2015	Shared decision-making	Physicians, scientists, patient advocacy groups, individual healthcare providers and members of the pharmaceutical industry	Patient, Physician	Clinical benefit Overall survival Progression-free survival Response rate Toxicity Bonus factor Palliation Time off all treatment Cost per month	Stakeholder consultation (ASCO Value in Cancer Care Task Force)	Net Health Benefit score and costs illustrated side by side to facilitate the decision-making process of patients by making fully informed decisions
ESMO (15)	2015	Clinical practice	ESMO Executive Board, members of the ESMO faculty, team of expert biostatisticians and a range of invited experts	Payer, Policymaker	Variability of estimated hazard ratio Observed absolute difference in treatment outcomes	Stakeholder consultation (ESMO Task Force with input from the ESMO faculty and a team of biostatisticians, followed by the ESMO MCBS Task Force, the ESMO Guidelines Committee and a range of invited experts)	Both the variability of the estimated hazard ratio (HR) and the observed absolute difference in treatment outcomes are explicitly addressed
MSKCC (17)	2015	Pricing	Physicians and scientists	Physician, Policymaker	Efficacy (survival) Toxicity Novelty Research and development Rarity Population health burden	Developed by a team of clinical experts	A range of domains incorporated, relating to both the drug and the disease
NCCN (18)	2015	Shared decision-making	Team of experts	Patient, Physician	Efficacy Safety Evidence quality Evidence consistency Affordability	Stakeholder consultation (NCCN panel members)	Easy and simple to comprehend visual output

Source: Schnipper, Davidson, Wollins, Blayney, Dicker, Ganz, et al. (2016), Cherny, Sullivan, Dafni, Kerst, Sobrero, Zielinski, et al. (2015), The Memorial Sloan Kettering Cancer Center (2016), National Comprehensive Cancer Network (2016).

**Table 2:** Steps in the implementation of Multi-Criteria Decision Analysis (MCDA), as recommended by the International Society For Pharmacoeconomics and Outcomes Research (ISPOR) Task Force for best practice in MCDA.

Step	Description
1. Defining the decision problem	Identify objectives, type of decision, alternatives, decision makers and output needed
2. Selecting and structuring criteria	Identify relevant criteria for assessing alternative technologies
3. Measuring performance	Add the performance data of the alternatives in the established criteria and summarize in a “performance matrix”
4. Scoring alternatives	Extracting the preferences of interest groups for performance variations in the criteria
5. Weighting criteria	Extracting the relative importance of the established criteria, based on the preferences of interest groups
6. Calculating aggregate scores	Use the criteria scores and the weights assigned to them to obtain the “total value”, through which the alternatives will be sorted
7. Dealing with uncertainty	Perform uncertainty analyses to understand the level of robustness of the results obtained
8. Reporting and examination of findings	Interpret analysis results, including uncertainty analyses, to support decision-making

Source: Marsh (2016).

## References

[1] Ferlay JI, Soerjomataram R, Dikshit S, Eser C, Mathers M, Rebelo DM (2015). Cancer incidence and mortality worldwide: sources, methods and major patterns in GLOBOCAN 2012. Int J Cancer.

[2] Ministério da Saúde (BR), Instituto Nacional de Câncer José Alencar Gomes da Silva (INCA), Coordenação de Prevenção e Vigilância (2015). Estimativa 2016: incidência de câncer no Brasil.

[3] Bach PB (2009). Limits on Medicare’s ability to control rising spending on cancer drugs. N Engl J Med.

[4] Neumann PJ, Cohen JT (2015). Measuring the value of prescription drugs. N Engl J Med.

[5] Boztepe S (2007). User value: competing theories and models. Int J Des.

[6] Gold MR, Siegel JE, Russell LB, Weinstein MC (1996). Cost-effectiveness in health and medicine.

[7] Brouwer WB, Culyer AJ, van Exel NJ, Rutten FF (2008). Welfarism vs. extra-welfarism. J Health Econ.

[8] Refoios Camejo R, Miraldo M, Rutten F (2017). Cost-effectiveness and dynamic efficiency: does the solution lie within?. Value Health.

[9] Garber AM, Phelps CE (1997). Economic foundations of cost-effectiveness analysis. J Health Econ.

[10] McCabe C, Claxton K, Culyer AJ (2008). The NICE cost-effectiveness threshold: what it is and what that means. Pharmacoeconomics.

[11] Sculpher M, Claxton K, Pearson SD (2017). Developing a value framework: the need to reflect the opportunity costs of funding decisions. Value Health.

[12] Perfetto EM, Oehrlein EM, Boutin M, Reid S, Gascho E (2017). Value to whom? The patient voice in the value discussion. Value Health.

[13] Sorenson C, Lavezzari G, Daniel G, Burkholder R, Boutin M, Pezalla E (2017). Advancing value assessment in the United States: a multistakeholder perspective. Value Health.

[14] Schnipper LE, Davidson NE, Wollins DS, Blayney DW, Dicker AP, Ganz PA (2016). Updating the American Society of Clinical Oncology value framework: revisions and reflections in response to comments received. J Clin Oncol.

[15] Cherny NI, Sullivan R, Dafni U, Kerst JM, Sobrero A, Zielinski C (2015). A standardised, generic, validated approach to stratify the magnitude of clinical benefit that can be anticipated from anti-cancer therapies: the European Society for Medical Oncology Magnitude of Clinical Benefit Scale (ESMO-MCBS). Ann Oncol.

[16] Institute for Clinical and Economic Review (2017). Overview of the ICER value assessment framework and update for 2017-2019. https://icer-review.org/methodology/icersmethods/icer-value-assessment-framework/.

[17] Memorial Sloan Kettering Cancer Center Drug Abacus FAQs.

[18] National Comprehensive Cancer Network NCCN clinical practice guidelines in oncology (NCCN Guideliness) with NCCN Evidence Blocks™.

[19] Mandelblatt JS, Ramsey SD, Lieu TA, Phelps CE (2017). Evaluating frameworks that provide value measures for health care interventions. Value Health.

[20] Malone DC, Berg NS, Claxton K, Garrison LP, IJzerman M, Marsh K (2016). International Society for Pharmacoeconomics and Outcomes Research Comments on the American Society of Clinical Oncology Value Framework. J Clin Oncol.

[21] Chandra A, Shafrin J, Dhawan R (2016). Utility of Cancer Value Frameworks for Patients, Payers, and Physicians. JAMA.

[22] Feeley TW, Fly HS, Albright H, Walters R, Burke TW (2010). A method for defining value in healthcare using cancer care as a model. J Healthc Manag.

[23] Bentley TG, Cohen JT, Elkin EB, Huynh J, Mukherjea A, Neville TH (2017). Validity and reliability of value assessment frameworks for new cancer drugs. Value Health.

[24] Michael FD, Mark JS, Karl C, Greg LS, George WT (2015). Methods for the Economic Evaluation of Health Care Programmes.

[25] Thokala P, Devlin N, Marsh K, Baltussen R, Boysen M, Kalo Z (2016). Multiple Criteria Decision Analysis for Health Care Decision Making—An Introduction: Report 1 of the ISPOR MCDA Emerging Good Practices Task Force. Value Health.

[26] Marsh PT, Devlin N, Marsh K, Baltussen R, Boysen M, Kalo Z (2016). Multiple Criteria Decision Analysis for Health Care Decision Making—Emerging Good Practices: Report 2 of the ISPOR MCDA Emerging Good Practices Task Force. Value Health.

[27] Campolina AG, Soárez PC, Amaral FV, Abe JM (2017). Análise de decisão multicritério para alocação de recursos e avaliação de tecnologias em saúde: tão longe e tão perto?. Cad Saúde Pública.

